# What is the duration of untreated psychosis worldwide?

**DOI:** 10.1017/S0033291724002125

**Published:** 2024-10

**Authors:** Nnamdi Nkire, Anthony Kinsella, Vincent Russell, John L. Waddington

**Affiliations:** 1 Cavan-Monaghan Mental Health Service, Drumalee Primary Care Centre, Cavan, Ireland; 2 School of Pharmacy and Biomolecular Sciences, RCSI University of Medicine and Health Sciences, Dublin, Ireland; 3 Department of Psychiatry, RCSI University of Medicine and Health Sciences, Beaumont Hospital, Dublin, Ireland; 4 Jiangsu Key Laboratory of Translational Research and Therapy for Neuro-Psychiatric-Disorders and Department of Pharmacology, College of Pharmaceutical Sciences, Soochow University, Suzhou, China

The association between increasing duration of untreated psychosis (DUP) and indices of poorer outcome (Howes et al., 2021) continues to exert a global influence on models of health care provision, including early intervention services for first-episode psychosis (FEP; Shah, Jones, van Os, McGorry, & Gülöksüz, 2022). A recent article ‘What is the duration of untreated psychosis worldwide’ (Salazar de Pablo et al., 2024) provides seminal information on differences in DUP across six global groupings (Africa, Asia, Australia, Europe, North America, South America) and indicates marked variation in DUP, both across continents and within regions, that requires explanation. The authors rightly emphasize socio-cultural determinants of health and differential pathways to care across diverse health care systems, but recognize also the potential impact of variations in how DUP is defined and what FEP diagnoses are included across studies. They address the challenge of varying diagnostic composition via sub-group analyses that involve exclusion of affective psychosis and of substance use disorders. Here, we propose first that this source of variation may have been underestimated, and second that the relationship between DUP and outcome, while robust, may be incomplete and in need of a broader concept to better understand the underlying processes.

To fully evaluate the impact of diagnostic composition on measures of DUP requires studies on epidemiologically representative populations that first incept *all* FEP diagnoses, apply standardized instruments to assess both diagnosis and DUP, and then progressively disassemble and compare these FEP diagnoses. The Cavan-Monaghan First Episode Psychosis Study (CAMFEPS; Nkire et al., 2021a) is a prospective study that sought to identify ‘all’ incident subjects presenting with FEP, without *a priori* diagnostic restriction, across two rural counties in Ireland. These contiguous counties share substantial socioeconomic and ethnic homogeneity, the vast majority of the population being native Irish, with no urban centers and minimal immigration. Cases were identified via all routes to care (public, private, and forensic; whether receiving home-based treatment or as outpatients or inpatients). All study features and assessments have been described previously in detail (Nkire et al., 2021a, 2021b).

Assessment of DUP was performed, to our knowledge for the first time, systematically across all 12 DSM-IV psychotic diagnoses (Nkire, Kinsella, Russell, & Waddington, 2024a). For convenience, these findings are consolidated in Table 1a–d in terms of four groupings of increasing diagnostic stringency, from all-inclusive, through more contemporary trans-diagnostic and spectrum concepts, to a more traditional, schizophrenia-centric approach: (a) any FEP diagnosis (*n* = 205); (b) a composite of schizophrenia spectrum and affective psychosis (*n* = 160); (c) schizophrenia spectrum psychosis (*n* = 71); (d) schizophrenia (*n* = 45).

**Table 1.** DPP, DUP, and DUI by diagnostic composition

Diagnostic composition		DPP	DUP	DUI
(a) Any DSM-IV psychotic diagnosis (*n* = 205)				
		12.7 (33.5)	6.7 (20.6)	19.4 (43.8)
		2.0 {0.5–9.0}	0.5 {0.0–3.1}	3.8 {1.0–15.0}
		[0–300]	[0–192]	[0–336]
(b) Schizophrenia spectrum and affective psychosis (*n* = 160)				
		13.5 (35.1)	7.2 (21.5)	20.7 (46.5)
		3.0 {0.5–9.8}	0.7 {0.0–3.9}	4.5 {1.0–15.0}
		[0–300]	[0–192]	[0–336]
(c) Schizophrenia spectrum psychosis (*n* = 71)				
		22.6 (47.9)	12.8 (27.6)	35.4 (62.4)
		5.0 {0.8–22.0}	3.0 {0.5–12.0}	12.0 {3.0–35.3}
		[0–300]	[0–192]	[0–336]
(d) Schizophrenia (*n* = 45)				
		31.5 (57.8)	18.3 (33.3)	49.8 (73.8)
		9.0 {0.9–34.0}	5.0 {1.8–24.0}	20.0 {6.0–63.5}
		[0–300]	[0–192]	[0–336]
(e) Bipolar disorder ‘with’ psychotic features (*n* = 41)				
		4.4 (13.2)	4.3 (19.7)	8.7 (24.5)
		1.0 {0.5–3.0}	0.2 {0.0–0.5}	1.1 {0.5–4.0}
		[0–84]	[0–120]	[0–132]
(f) Bipolar disorder ‘without’ psychotic features (*n* = 10)				
		2.8 (3.4)	0.3 (0.4)	3.1 (3.4)
		1.3 {0.8–4.5}	0.1 {0.0–0.5}	1.8 {0.8–4.9}
		[0–9]	[0–1]	[0–9]

(a) Any DSM-IV psychotic diagnosis: schizophrenia (SZ); schizophreniform disorder (SF); brief psychotic disorder; schizoaffective disorder (SA); bipolar I disorder (BD) [(e) BD ‘with’ and (f) BD ‘without’ psychotic features]; major depressive disorder with psychotic features (MDDP); delusional disorder; substance-induced psychotic disorder; substance-induced mood disorder, with manic features; psychotic disorder due to a general medical condition; mood disorder due to a general medical condition, with manic features; and psychotic disorder not otherwise specified. (b) Composite of schizophrenia spectrum and affective psychosis: SZ, SF, SA, BD, and MDDP. (c) Schizophrenia spectrum psychosis: SZ, SF, and SA. (d) SZ. DPP, duration of the psychosis prodrome; DUP, duration of untreated psychosis; DUI, duration of untreated illness = DPP + DUP. Data for DPP, DUP, and DUI are number of cases, mean (s.d.), median {interquartile range} and range [minimum–maximum] in months. For details on individual diagnoses see Nkire et al. (2024a, 2024b).

Across these four groupings increasing diagnostic stringency was associated with a marked ordinal increase in DUP, threefold by mean and 10-fold by median, that is likely to vary with both time and space: with time due to a traditionally schizophrenia-centric concept of psychotic illness that has progressively broadened to contemporary, transdiagnostic concepts of real-world FEP; with space due to some latency in these contemporary concepts generalizing from Australia, Europe, and North America to Africa, Asia, and South America. The magnitude of variation in median DUP associated with increasing diagnostic stringency (in CAMFEPS 10-fold; Table 1a–d) appears considerably greater than that evident across continents (2.8-fold).

Salazar de Pablo et al. note modest (1.4-fold) variation in global estimates of median DUP with *v*. without exclusion of affective psychosis. Disassembly of these groupings in CAMFEPS indicates that, relative to schizophrenia, DUP is substantially shorter for bipolar disorder and intermediate for major depressive disorder with psychotic features (Nkire et al., 2024a). Thus, the magnitude of variation in median DUP associated with the inclusion of neither, either, or both of bipolar disorder and major depressive disorder with psychotic features in a given FEP study appears substantial.

As an additional cause of variation in DUP worldwide, FEP studies that encompass affective psychosis typically include bipolar disorder on the basis of only one or both of two clinically based subtypes: ‘with’ *v*. *‘*without’ psychotic features (Aminoff et al., 2022). In CAMFEPS the incidence of bipolar disorder ‘with’ psychotic features is 2.5-fold higher than for ‘without’ such features and these two groups are indistinguishable in terms of age, sex, and assessments of psychopathology, neuropsychology, neurology, movement disorder, premorbid features, insight, and quality of life (Nkire, Kinsella, Russell, & Waddington, 2024b). We now report that these two putative subtypes are also indistinguishable in terms of DUP (Table 1e, f). This elaborates our proposition that these two groups reflect dichotomization at a subjective threshold along a continuously distributed dimension of psychosis severity that is intrinsic to bipolar disorder (Nkire et al., 2024b).

Salazar de Pablo et al. also note modest (1.6-fold) variation in global estimates of median DUP with *v*. without exclusion of FEP with substance use disorders. In CAMFEPS substance-induced psychotic disorder has a multi-fold shorter median DUP relative to schizophrenia (Nkire et al., 2024a), further emphasizing the extent to which diagnostic composition can influence estimates of DUP across FEP studies.

Preceding DUP is the phase of clinical high risk for psychosis (CHR-P) and putative interventions to reduce transition from CHR-P to FEP (Shah et al., 2022). However, the historical record back to 1881 (see Nkire et al., 2024a; Nkire, Kingston, Kinsella, Russell, & Waddington, 2023) emphasizes first noticeable symptoms that begin considerably prior to identification of CHR-P to augur the hallucinations and delusions of psychotic illness. The interval from such first noticeable symptoms to the start of DUP is duration of the psychosis prodrome (DPP) and poses a fundamental question: are DPP and DUP two independent constructs, each having distinct characteristics, or two successive components of the same construct, each having similar characteristics?

We have recently studied the quantitative characteristics of DPP for each of the 12 DSM-IV psychotic diagnoses and find the rank order for DPP across these diagnoses to be essentially identical to that for DUP (Nkire et al., 2024a). These findings are consolidated and juxtaposed with those for DUP in Table 1a–d for the same diagnostic groupings. Notably, DPP was invariably longer than DUP and medians for DPP and DUP were invariably shorter than their means, indicating common right-skewed distributions. These findings indicate each of DPP and DUP, together with their consolidation in duration of untreated illness (DUI = DPP + DUP; Table 1a–d), to share very similar quantitative characteristics. In terms of prognostic significance, DUP and DUI predict both negative symptom severity and poorer quality of life at FEP with very similar regression coefficients (Nkire et al., 2021b), indicating common rates of change in each outcome measure per unit change in each duration.

In summary, Salazar de Pablo et al. cogently emphasize socio-cultural determinants of health and differential pathways to care across diverse health care systems as important explanatory factors for variation in estimates of DUP. However, the impact of variation in diagnostic practice is likely to have been underestimated. Furthermore, perspectives on DUP may benefit from a more holistic concept of DPP and DUP as reflecting dichotomization at a subjective threshold along a common underlying process. This might be better quantified as duration of untreated illness, with attendant revision of their individual *v*. collective relationships with outcome.
